# Patient characteristics, short-term and long-term outcomes after incident heart failure admissions in a regional Australian setting

**DOI:** 10.1136/openhrt-2021-001897

**Published:** 2022-05-31

**Authors:** Mohammed S Al-Omary, Tazeen Majeed, Hafssa Al-Khalil, Stuart Sugito, Mathew Clapham, Doan T M Ngo, John R Attia, Andrew J Boyle, Aaron L Sverdlov

**Affiliations:** 1 Cardiovascular Department, John Hunter Hospital, New Lambton Heights, New South Wales, Australia; 2 College of Health, Medicine and Wellbeing, University of Newcastle, Newcastle, New South Wales, Australia; 3 The University of Newcastle, Callaghan, New South Wales, Australia; 4 John Hunter Hospital, New Lambton Heights, New South Wales, Australia; 5 Hunter Medical Research Institute, Newcastle, New South Wales, Australia

**Keywords:** Heart Failure, Outcome Assessment, Health Care, Medication Adherence, Quality of Health Care

## Abstract

**Aims:**

This study aims to (1) define the characteristics of patients with a first admission for heart failure (HF), stratified by type (reduced (HFrEF) vs preserved (HFpEF) ejection fraction) in a regional Australian setting; (2) compare the outcomes in terms of mortality and rehospitalisation and (3) assess adherence to the treatment guidelines.

**Methods:**

We identified all index hospitalisations with HF to John Hunter Hospital and Tamworth Rural Referral Hospital in the Hunter New England Local Health District over a 12 months. We used the recent Australian HF guidelines to classify HFrEF and HFpEF and assess adherence to guideline-directed therapy. The primary outcome of the study was to compare short-term (1 year) and long-term all-cause mortality and the composite of all-cause hospitalisation or all-cause mortality of patients with HFrEF and HFpEF.

**Results:**

There were 664 patients who had an index HF admission to John Hunter and Tamworth hospitals in 2014. The median age was 80 years, 47% were female and 22 (3%) were Aboriginal. In terms of HF type, 29% had HFrEF, 37% had HFpEF, while the remainder (34%) did not have an echocardiogram within 1 year of admission and could not be classified. The median follow-up was 3.3 years. HFrEF patients were predominantly male (64%) and in 48% the aetiology was ischaemic heart disease. The 1-year all-cause mortality was 23% in HFpEF subgroup and 29% in HFrEF subgroup (p=0.15). Five-year mortality was 61% in HFpEF and HFrEF patients. Of the HFrEF patients, only 61% were on renin-angiotensin-aldosterone blockers, 74% were on β-blockers and 39% were on aldosterone antagonist.

**Conclusion:**

HF patients are elderly and about evenly split between HFrEF and HFpEF. In this regional cohort, both HF types are associated with similar 1-year and 5-year mortality following incident HF hospitalisation. Echocardiography and guideline-directed therapies were underused.

**WHAT IS ALREADY KNOWN ON THIS TOPIC**

- Heart failure (HF) is associated with significant morbidity and mortality. Use of guideline medical therapy in important to improve short-term and long-term outcomes.

**WHAT THIS STUDY ADDS**

- Short-term and long-term outcomes of HF with reduced ejection fraction (HFrEF) and HF with preserved ejection fraction in Australia and the limited use of echocardiography and medical therapy in HF patients.

**HOW THIS STUDY MIGHT AFFECT RESEARCH, PRACTICE AND/OR POLICY**

- This study confirmed the burden of HF types in the regional communities. It highlights the importance of incident HF admission and the associated high mortality following this admission. In addition, its critical importance of adherence to guideline medical therapy in HFrEF and the need for appropriate use of echocardiography. In addition, these results will help policymakers to appropriately allocate more resources not only to regional Australia; but also to regional and rural communities in other countries in Europe and North America due to similarities in health systems. The study provides an important insight into HF management and represents a real-life experience that is likely to be happening worldwide.

## Introduction

Heart failure (HF) is a major health problem and is associated with high rates of mortality and morbidity.^1^ Despite advances in treatment options, HF hospitalisation rates have been steady, if not increasing, over the last decade.^2^ The most recent Australian HF guidelines, published in 2018, divide HF into two main types: HF with reduced ejection fraction (HFrEF) and HF with preserved ejection fraction (HFpEF).^3^ HFpEF is defined as clinical symptoms and signs of HF and EF of at least 50%, while HFrEF is diagnosed if EF is less than 50%.^3^ While there are proven therapeutic options to reduce mortality and morbidity in HFrEF, no such options existed for HFpEF,^3 4^ until the results of EMPEROR-Preserved trial were reported last month.^5^

There is a discrepancy in the literature regarding the outcomes for different types of HF. While data from the US registry and smaller Japanese prospective cohort^6^ suggest that HFrEF and HFpEF have both similar prevalence and similar patient outcomes,^7^ contemporary data from a prospective Italian study^8^ and a registry from California^9^ showed higher mortality in HFrEF compared with HFpEF patients. There is a paucity of data reporting HFpEF and HFrEF outcomes in Australia, and most Australian HF studies reported outcomes on HF overall, rather than by HF type.^10–12^ The recent HF Snapshot study was one of the few studies that reported outcomes by HF type.^13^ Furthermore, despite cardiovascular death rates being 1.2 and 1.3 times higher in areas defined as regional (170 deaths/100 000) or remote (194/100 000) compared with major cities (147/100 000) in Australia, almost no data exist about HF outcomes in these populations.^14 15^ The current study aims to characterise the main two types of HF in a regional Australian setting, compare the outcomes between these two groups in terms of mortality and rehospitalisation, and assess adherence to treatment guidelines in HFrEF. This would help identify current practice gaps and inform future policy interventions to improve HF management in regional Australia.

## Methods

The Hunter New England region of New South Wales, Australia, covers an area of over 130 000 km^2^, and has a population of approximately 910 000, of whom approximately 45% live in metropolitan areas and 55% in regional or rural settings. John Hunter Hospital is the only major tertiary metropolitan teaching hospital in the district. Tamworth Rural Referral Hospital is the main inland Hospital that covers a large catchment of remote areas in the district. Approximately 15% of the population were born overseas and about 5% of the population are Aboriginal and Torres Strait Islanders.^16^

We identified all index hospitalisations with HF to John Hunter and Tamworth Rural Referral Hospitals in the Hunter New England Local Health District over 12 months (January to December 2014). John Hunter Hospital is located in Newcastle but serves a population of just over 0.5 million in the Greater Newcastle Area, classified as Australian Statistical Geographical Classification-Remoteness Area (ASGC-RA) one (major city); Tamworth is classified as ASGC remoteness category RA2 (inner regional). To identify *index* HF admissions only, patients with a prior admission with HF within the preceding 10 years (from January 2005 to December 2013) were excluded. Records with an ICD10 (International Statistical Classification of the Diseases and related health problems) code for clinically diagnosed HF (I-50) as a principal diagnosis or one of the first three secondary diagnoses on discharge were extracted and linked to the NSW state registry of births and deaths. Patient presentations to emergency departments not resulting in admission and patients younger than 15 years old were excluded. Comorbidities were identified from medical chart extraction during the index hospitalisation. Precipitants for the HF admission, aetiology of HF, admission service and HF types were extracted from medical records (see online supplemental data). A sensitivity analysis was performed using HF as a principal diagnosis. Data on medications at admission and discharge were also extracted (see online supplemental data). Echocardiographic ejection fraction was collected if the participant had echocardiography within 1 year of admission (6 months before or 6 months after). Patients who died during index hospitalisation were excluded from the analysis of readmission. Data on outcomes were collected from the time of index admission till December 2020.

10.1136/openhrt-2021-001897.supp1 Supplementary data

The primary aim of the study was to compare 1 year and long-term all-cause mortality and the composite of all-cause hospitalisation or all-cause mortality of patients with HFrEF and HFpEF. The secondary endpoints were comparisons of demographics, comorbidities, aetiologies, precipitants of HF admission, 1-year cardiovascular readmission rates, 1-year all-cause readmission rates, 1-year second all-cause readmissions and outcomes according to admitting specialty between HFrEF and HFpEF patients. We also compared outcomes between HFrEF patients who were on guideline-compliant medical management and those not on guideline-compliant medical therapy (ACE inhibitor/angiotensin II receptor blockers and β-blocker and/or aldosterone antagonists). These three agents were recommended by the contemporary American and Australian guidelines in patients with HFrEF and EF<40%.^17 18^

Propensity scores, using the stabilised inverse probability of treatment weighting (IPTW) method, were used to account for differences between HFrEF and HFpEF in the logistic and Cox regressions analyses. The propensity scores were estimated using a logistic regression on HF groups with the following covariates: age, gender, hypertension, ischaemic heart disease, chronic kidney disease, history of bypass, haemoglobin, haematocrit, white blood cell, neutrophil count, lymphocyte count, monocyte count,^19^ estimated glomerular filtration rate and diuretic use (see online supplemental file for more details). The calculated stabilised weights were used in the final regression models with the outcomes of 1-year all-cause mortality (expressed as OR), time to death and time to readmission (expressed as HR). A Cox regression including death as a competing risk with IPTW was used to assess the difference in time from admission to first readmission and first HF readmission between HF groups.

Data extraction was performed by four investigators (MSA-O, HA-K, SS) and interobserver variability was assessed to ensure accuracy. We used a mean±SD or median and IQR (as appropriate) to summarise continuous variables, and numbers and percentages for binary variables. We used the χ^2^ test to compare binary variables and parametric and non-parametric tests, as appropriate, to compare continuous variables. Simple and multiple models for predictors of 1-year all-cause mortality were constructed using logistic regression. For HFpEF and HErEF patients, simple and multiple models for predictors of long-term all-cause mortality were constructed using Cox regression. The first model includes all variables with p<0.2 on univariate analyses, whereas the further models include only variables with p<0.05, via stepwise deletion. We used p<0.05 as the level of significance. Data were analysed using STATA/SE V.14.1 (Stata Corp) and SAS V.9.4 (SAS Institute).

## Results

### HF cohort

In 2014, there were 664 patients with an index HF admission to John Hunter or Tamworth hospitals. The median age was 80 years (IQR 71–86), 311 (47%) were female and 22 (3%) were Indigenous. In terms of HF types, 193 patients (29%) had EF<50% and were classified as HFrEF, 247 (37%) had EF≥50% and were classified as HFpEF, while the remaining 224 patients (34%) did not have a recorded EF (non-classified HF). The majority (90%) of echocardiograms were performed during the index hospitalisation. The main demographic features, precipitant for admission, aetiology of HF and associated comorbidities (see table 1). The diagnosis of HF was clinical by the attending physician and 266 patients (40%) had HF as principal diagnosis. The overall result from the sensitivity analysis (HF as the principal diagnosis rather than in the top four) was similar to that from the entire cohort (see online supplemental file). The median follow-up was 3.3 years. Coronary angiography was performed in 73 patients (11%). Of those, 36 underwent percutaneous coronary intervention (5%). Valve replacement surgery was performed in 46 patients (7%). Only one patient had cardiac resynchronisation therapy.

**Table 1:** Baseline demographics, comorbidities, cause of HF, precipitants of HF admission and specialty of admission according to HF type

	HFrEF (n=193)	HFpEF (n=247)	Non-classified HF (n=224)	Total (n=664)	P value
Demographics					
Age in years, median (IQR)	77 (66–84)	79 (71–85)	82 (75–88.5)	80 (71–86)	<0.001
Female, number (%)	65 (34)	128 (52)	118 (53)	311 (47)	<0.001*
Indigenous, number (%)	9 (5)	10 (4)	3 (1)	22 (3)	0.12
Rural, number (%)	94 (49)	98 (40)	76 (34)	268 (40)	0.009
Length of stay in days, (IQR)	6 (3–10)	6 (3–11)	4 (2–8)	5 (3–10)	<0.001
Precipitant for admission, number (%)					
Adherence	11 (6)	11 (4)	17 (8)	39 (6)	0.34
Arrhythmia	39 (20)	44 (19)	42 (19)	125 (19)	0.81
Ischaemia	52 (27)	46 (19)	24 (11)	122 (18)	<0.001*
Infection	44 (23)	69 (28)	75 (33)	188 (28)	0.053
Other/unknown	68 (35)	93 (38)	81 (36)	242 (36)	0.86
Cause of HF, number (%)					
IHD	109 (56)	106 (43)	105 (47)	320 (48)	0.016*
Cardiomyopathy	32 (17)	16 (6)	16 (7)	64 (10)	0.001*
VHD	28 (15)	47 (19)	32 (14)	107 (16)	0.29
Comorbidities, number (%)					
Atrial fibrillation/flutter	84 (44)	113 (46)	102 (47)	299 (45)	0.86
Stroke	23 (12)	39 (16)	42 (19)	104 (16)	0.148
CPD	55 (29)	81 (33)	68 (31)	204 (31)	0.608
CKD	57 (30)	103 (42)	68 (31)	228 (35)	0.009*
Diabetes mellitus	62 (32)	73 (30)	59 (27)	194 (29)	0.47
Hypertension	105 (55)	179 (72)	138 (62)	422 (64)	0.001*
IHD	112 (58)	111 (45)	107 (48)	330 (50)	0.02*

*Comparison between HFpEF and HFrEF is significant at p value <0.05 level.

CKD, chronic kidney disease; CPD, chronic pulmonary disease; HF, heart failure; HFpEF, heart failure with preserved ejection fraction; HFrEF, heart failure with reduced ejection fraction; IHD, ischaemic heart disease; VHD, valvular heart disease.

### Primary outcomes

The rate of 1-year all-cause mortality HFrEF (29%) and HFpEF (23%) (p=0.15). Similarly, 5-year all-cause mortality was in HFrEF and HFpEF (61%) (p=0.9). The composite of 1-year all-cause readmission or mortality was 67% (n=446); this was 66% in HFpEF, 66% in HFrEF and 69% in non-classified HF (p=0.72); and 94% of patients had either death or readmission within 5 years of index admission (see table 2).

**Table 2:** Discharge medication, specialty of admission, pathology result and outcomes according to HF type

	HFrEF (n=193)	HFpEF (n=247)	Non-classified HF (n=224)	Total (664)	P value*
Discharge medication, number (%)					
ACEi/ARB	118 (61)	133 (55)	110 (50)	361 (55)	0.07
β-blockers	142 (74)	145 (61)	123 (56)	410 (63)	0.001†
Aldosterone antagonist	74 (39)	64 (27)	39 (18)	177 (27)	<0.001†
Anticoagulant	68 (36)	77 (32)	60 (28)	205 (32)	0.22
Antiplatelet agents	116 (60)	124 (50)	95 (42)	335 (50)	0.002†
Calcium channel blocker	24 (13)	38 (16)	24 (11)	86 (13)	0.26
Digoxin	30 (16)	32 (14)	40 (18)	102 (16)	0.41
Loop diuretics	148 (78)	170 (69)	147 (66)	465 (70)	0.043
Thiazide diuretics	6 (3)	23 (9)	10 (4)	39 (6)	0.013†
Nitrate	23 (12)	23 (10)	16 (7)	62 (10)	0.28
Statins	101 (52)	133 (55)	85 (39)	319 (49)	0.001
Cardiac rehab	57 (30)	62 (25)	22 (10)	141 (21)	<0.001
Specialty, number (%)					
Cardiology	98 (51)	94 (38)	57 (25)	249 (38)	
OMUs	95 (49)	153 (62)	167 (75)	415 (62)	<0.001†
Pathology					
Peak troponin in µg/L, median (IQR)	67 (25–1384)	41 (15–223)	41 (18–157)	49 (20–245)	0.008†
Haemoglobin in g/L, mean (SD)	126 (20)	120 (21)	122 (21)	122 (21)	0.024†
White blood cell ×10^9^/L, median (IQR)	9 (7–13)	9 (7–12)	9.6 (8–13)	9 (7–12)	0.008
Platelet ×10^9^/L, median (IQR)	210 (160–266)	223 (173–289)	213 (169–272)	215 (169–278)	0.21
Albumin in g/L, mean (SD)	37 (4)	37 (4)	36 (5)	36 (4)	0.3
GFR in mL/min/1.73m^2^, median (IQR)	63 (42–78)	53 (35–71)	56 (35–75)	56 (37–75)	0.008†
Outcomes, number (%)					
30-day death	19 (10)	14 (6)	50 (22)	83 (12.5)	<0.001
1-year death	56 (29)	57 (23)	84 (38)	197 (30)	0.003
1-year all-cause readmission or mortality	127 (66)	164 (66)	155 (69)	446 (67)	0.72
30-day all-cause readmission	29 (15)	50 (20)	30 (13)	109 (16)	0.11
1-year all cause readmission	98 (51)	145 (59)	94 (42)	337 (51)	0.001
CV readmission	47 (47)	53 (36)	27 (29)	127 (38)	0.028
Second all-cause readmission	49 (25)	84 (34)	48 (21)	181 (27)	0.007
5-year all-cause death	117 (61)	151 (61)	161 (72)	439 (65)	0.02
5-year all-cause readmission	144 (75)	206 (83)	147 (66)	497 (75)	<0.001†
5-year HF readmission	79 (41)	118 (48)	70 (31)	267 (40)	0.001

*P value represents the comparison between the three heart failure groups.

†Comparison between HFpEF and HFrEF is significant at p value <0.05 level

ACEi/ARB, ACE inhibitor/angiotensin II receptor blockers; CV, cardiovascular; GFR, glomerular filtration rate; OMUs, other medical units.

### Secondary outcomes

#### HF types and demographics, precipitant of admission and co-morbidities

The median age was 77 years in HFrEF and 79 years in HFpEF (p=0.09). Non-classified HF patients had the shortest length of stay compared with HFrEF and HFpEF (median of 4 days vs 6 days and 6 days, respectively; p<0.001). The most common known precipitant of HF admission was an infection in HFpEF (28%), while myocardial ischaemia was the most common precipitant in HFrEF (27%). Hypertension was more common in HFpEF (72%) compared with HFrEF (55%) (p=0.001). There were significant differences between HFrEF and HFpEF in the frequency of chronic kidney disease and ischaemic heart disease (p<0.001, p=0.008, p=007, respectively; table 1).

Our cohort included 311 (47%) female patients with an index HF admission. Female patients were older (mean of 81 vs 79 years, p=0.002), had less ischaemic heart disease (40% vs 59%, p<0.001) and less chronic lung disease (27% vs 35%, p=0.02) than males. Female HF patients were more likely to have HFpEF (41% vs 34%) and less likely to have HFrEF (21% vs 36%) (p<0.001). There was no gender difference in 1-year and 5-year all-cause mortality or readmissions (1 year: 30% vs 29% and 50% vs 51%, respectively, 5 years: 66% vs 63% and 74% vs 76%, respectively).

#### Discharge medication in HF and guideline medical therapy in HFrEF

**Figure 1:**
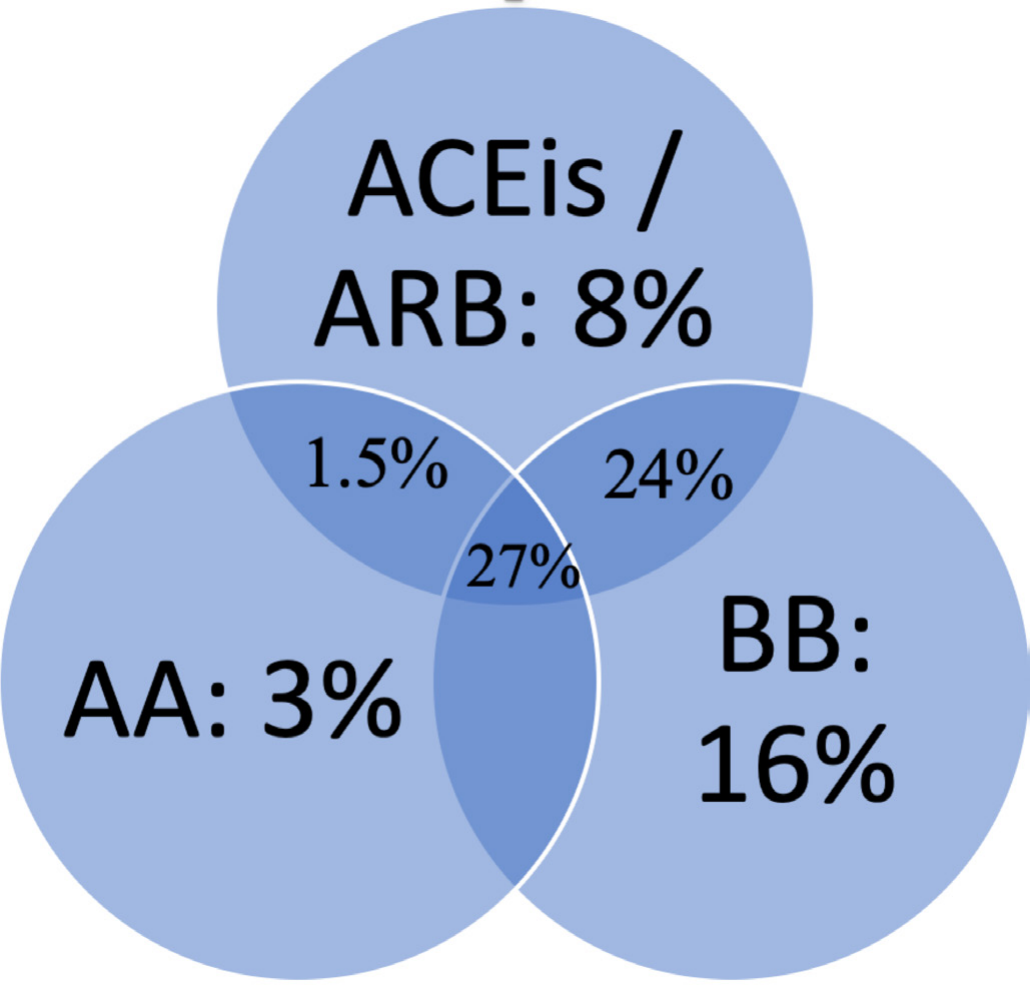
Medical guideline therapy in HFrEF. 14% were not on ACEis/ARB, BB or AA. AA, aldosterone antagonist; ACEis/ARB, ACE inhibitors/angiotensin II receptor blockers; BB, β-blockers; HFrEF, heart failure with reduced ejection fraction.

When we used 2011 Australian guidelines, (HFrEF definition: EF≤40%), of the 145 HFrEF patients, 90 patients (62%) were on ACE inhibitor/angiotensin II receptor blockers, 112 (77%) were on β-Blockers and 58 (40%) were on an aldosterone antagonist. However, when we apply the new 2018 guidelines, the number of HFrEF patients increased to 193 patients, of which 118 (61%) were on ACE inhibitor/angiotensin II receptor blockers, 142 (74%) were on β-Blockers, 74 (39%) were on aldosterone antagonist and 148 (78%) were on loop diuretics (figure 1). However, only 100 patients (52%) were on the combination of ACE inhibitor/angiotensin II receptor blockers and β-Blockers, and only 53 (27%) were triple therapy. HFrEF patients on the combination of both renin-angiotensin-aldosterone inhibitors and β-blockers had significantly lower unadjusted 1-year and 5-year all-cause mortality compared with HFrEF patients who were on neither or only on one of the agents (1 year: 19% vs 40%, p=0.001, 5 years: 50% vs 67%, p=0.002). Similarly, HFrEF patients on the combination of all three classes of medications: ACE inhibitor/angiotensin II receptor blockers, β-blockers and aldosterone antagonists, had significantly lower 1-year and 5-year all-cause mortality compared with HFrEF patients who were on none or only one class of HF medications (1 year: 17% vs 34%, p=0.023, 5 years: 40% vs 69%, p<0.001). There was a significant increase in the prescription of β-blockers and aldosterone antagonist on discharge but not ACE inhibitor/angiotensin II receptor blockers, compared with admission, noted in the sensitivity analysis (see online supplemental file).

#### Admitting specialty

Of the 664 patients, 249 patients were admitted under cardiology, while 388 patients were under other medical units (OMU), with the majority of these admissions under general medicine specialty. Patients under OMU were older (median age of 81 vs 78 years, p<0.001) and had longer hospitalisation (median 6 vs 5 days, p=0.023). Ischaemic heart disease was the most common comorbidity in cardiology-admitted patients 57% versus 45% (p=0.003), while chronic kidney disease and chronic respiratory disease were more prevalent in OMU-admitted HF patients (38% vs 30%, p=0.04 and 36% vs 24%, p=0.002, respectively). Admission under OMU was a predictor of 1-year mortality on univariate analysis (OR 2.3, 95% CI 1.6 to 3.4, p<0.001); but this dropped out after adjustment for other covariates (OR 1.4, 95% CI 0.85 to 2.25, p=0.19; table 3). The ACE inhibitor/angiotensin II receptor blockers, β-blockers and aldosterone antagonists were prescribed more frequently in cardiology-admitted patients (67% vs 49%; 80% vs 55%; and 44% vs 17%, respectively: p<0.001).

**Table 3:** Univariate and multivariate predictors of 1-year all-cause mortality

Variable	Univariate analysis			Multivariate analysis*		
	OR	95% CI	P value	OR	95% CI	P value
Demographics						
Age	1.04	1.03.1.06	<0.001	1.04	1.02 to 1.06	<0.001
Female	1.02	0.73 to 1.4	0.9	-–	–	–
Indigenous	1.1	0.4 to 2.7	0.82	–	–	–
Rural	1.4	1.01 to 1.9	0.047	1.6	1.06-.25	0.026
Precipitant of admission						
Adherence	1.05	0.5 to 2.1	0.87	–	–	–
Arrhythmia	0.3	0.2 to 0.6	<0.001	0.5	0.3 to 0.9	0.04
Ischaemia	0.7	0.5 to 1.2	0.25	–	–	–
Infection	1.2	0.8 to 1.7	0.32	–	–	–
Cause of HF						
IHD	1.03	0.7 to 1.4	0.85	–	–	–
Cardiomyopathy	0.6	0.3 to 1.1	0.154	–	–	–
VHD	1.3	0.8 to 2.1	0.14	–	–	–
Comorbidities						
Atrial fibrillation/flutter	0.86	0.6 to 1.2	0.39	–	–	–
Stroke	1.7	1.1 to 2.6	0.016	1.9	1.1 to 3.2	0.021
CPD	1.3	0.9 to 1.9	0.08	–	–	–
CKD	1.7	1.2 to 2.4	0.002	0.9	0.6 to 1.7	0.97
Diabetes mellitus	0.84	0.5 to 1.2	0.36	–	–	–
Hypertension	0.7	0.5 to 1.01	0.058	–	–	–
IHD	1.09	0.78 to 1.53	0.59	–	–	–
Specialty						
OMUs	2.34	1.6 to 3.4	<0.001	1.4	0.9 to 2.3	0.18
HF type (compared with unknown)						
HFrEF (EF<50)	0.68	0.4 to 1.02	0.068	1.3	0.8 to 2.1	0.37
HFpEF (EF≥50)	0.5	0.3 to 0.7	0.001	0.6	0.4 to 0.99	0.05
Medication						
ACEis/ARB	0.38	0.27 to 0.54	<0.001	0.5	0.3 to 0.8	0.002
Aldosterone antagonist	0.59	0.39 to 0.88	0.01	1.4	0.8 to 2.3	0.25
β-Blockers	0.36	0.25 to 0.5	<0.001	0.6	0.4 to 0.98	0.044
Anticoagulant	0.52	0.35 to 0.77	0.001	0.7	0.4 to 1.2	0.19
Antiplatelets	0.6	0.4 to 0.8	0.006	0.7	0.4 to 1.1	0.11
Calcium channel blocker	0.73	0.4 to 1.2	0.25	–	–	–
Digoxin	0.4	0.2 to 0.7	0.005	0.5	0.2 to 0.9	0.02
Loop diuretics	0.6	0.4 to 0.86	0.006	0.94	0.6 to 1.5	0.79
Thiazide	0.5	0.2 to 1.3	0.201	–	–	–
Nitrate	1.05	0.6 to 1.8	0.84	–	–	–
Statins	0.4	0.34 to 0.68	<0.001	0.8	0.5 to 1.2	0.24
Cardiac rehab	0.4	0.25 to 0.67	<0.001	0.8	0.5 to 1.5	0.57
Pathology						
Peak troponin	1	0.9 to 1.1	0.65	–	–	–
Haemoglobin	0.98	0.97 to 0.99	<0.001	1	0.9 to 1.01	0.38
White blood cell	0.9	0.9 to 1.01	0.5	–	–	–
Platelet count	0.99	0.9 to 1	0.169	–	–	–
Albumin	0.9	0.86 to 0.93	<0.001	0.93	0.89 to 0.98	0.009
GFR at admission	0.98	0.97 to 0.99	<0.001	0.99	0.98 to 1.01	0.25
Creatinine at admission	1.01	1.01 to 1.02	0.008	–	–	–

*Model included variables with p value of less than 0.05.

ACEis/ARB, ACE inhibitors/angiotensin II receptor blockers; CKD, chronic kidney disease; CPD, chronic pulmonary disease; GFR, glomerular filtration rate; HF, heart failure; IHD, ischaemic heart disease; OMUs, other medical units; VHD, valvular heart disease.

#### Propensity matched scores and univariable and multivariable predictors of mortality

The IPTW method increased the balance of selected covariates across HF groups and showed no significant difference between HFpEF and HFrEF groups (online supplemental file).

**Figure 2:**
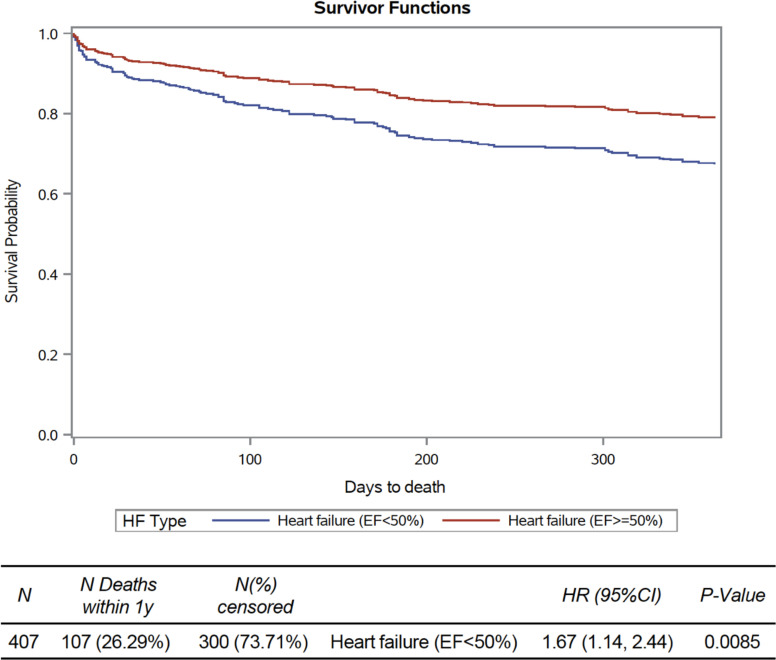
Propensity matched-score survival probability. HR of 1.67 (95% CI 1.14 to 2.44, p=0.008). EF, ejection fraction; HF, heart failure.

There were 107 (26%) patients with non-missing covariates that died within 1 year. Using IPTW method, patients in HFrEF group had 67% higher 1-year all-cause mortality than HFpEF group (HR=1.67, 95% CI 1.14 to 2.44; p=0.009) (figure 2).

There were 267 (66%) patients with non-missing covariates that died or were readmitted at least once within 1 year. There was no difference in death or readmission within 1 year between the HFrEF and HFpEF groups (adjusted OR 1.17, 95% CI 0.78 to 1.76; p=0.45). There were 216 (53%) patients with non-missing covariates that were readmitted within 1 year, and 51 (13%) had competing events (death within 1 year). The adjusted HR for readmission was not different between HFrEF and HFpEF groups (0.81, 95% CI 0.62 to 1.06; p=0.13). Similarly, there was no significant difference between HFrEF and HFpEF patients with regards to cardiovascular readmissions (HR 0.75, 95% CI 0.53 to 1.07, p=0.12; table 2 and online supplemental file).

The univariate predictors and multivariable analysis of 1-year all-cause mortality are shown in table 3. In multivariate model 1, age (OR 1.04, p<0.001), rurality (OR 1.6, p=0.027) and history of stroke (OR 1.8, p=0.03) were predictors of poor outcome. However, admission for arrhythmia (OR 0.5, p=0.045), ACE inhibitor/angiotensin II receptor blockers use (OR 0.35, p=0.006), digoxin use (OR 0.4, p=0.013) and high albumin (OR 0.9, p=0.019) predicted lower 1-year all-cause mortality. The second multivariable model using variables with p<0.05 showed similar results to the first model. In addition, β-blockers use on discharge predicted lower 1-year mortality (OR 0.6, p=0.04; table 3). Interestingly, similar to propensity matched score results, HFpEF showed a trend of lower 1-year mortality risk (OR 0.6, p=0.05).

#### Long terms outcomes and Cox regression

**Figure 3:**
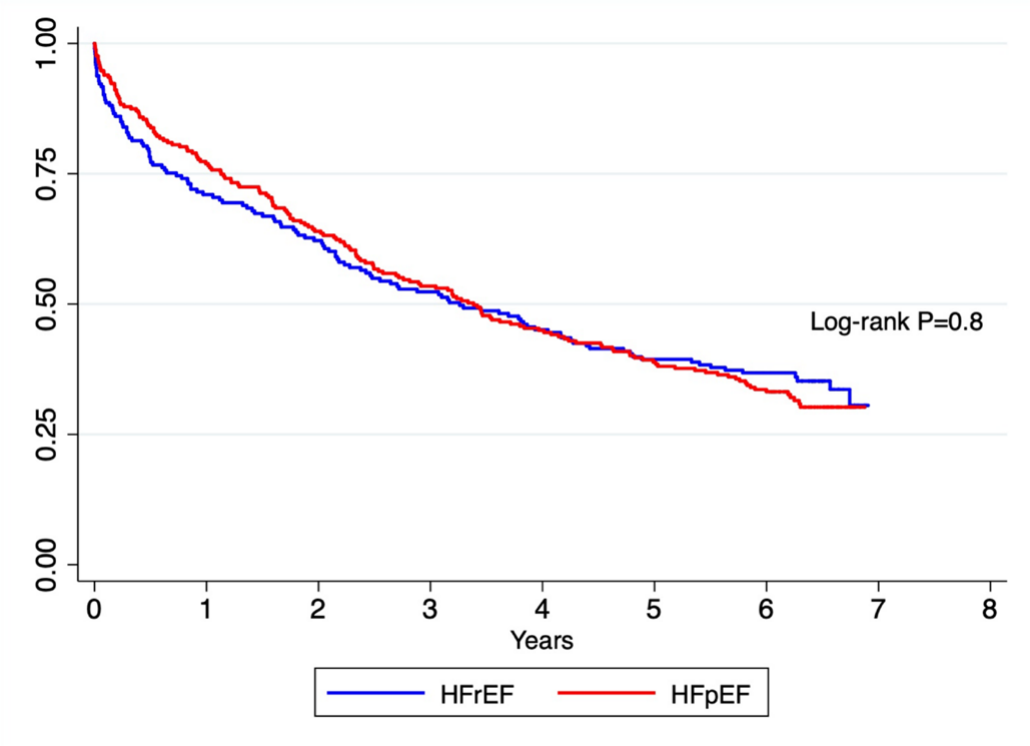
Long-term all-cause mortality of HFpEF and HFrEF patients. HFpEF, heart failure with preserved ejection fraction; HFrEF, heart failure with reduced ejection fraction.

The 5-year all-cause mortality was 65%, 5-year all-cause readmission was 75% and the composite all-cause readmission or mortality was 94%. Five-year HF-readmission was 40% (table 2 and figure 3). For HFpEF and HFrEF patients, older age (HR 1.05, p<0.001) and elevated creatinine on admission (HR=1.002, p=0.01) were predictors of long-term mortality. However, being on ACE inhibitor/angiotensin II receptor blocker (HR=0.7, p=0.027) and higher albumin on admission (HR=0.95, p=0.005) were predictors of better long-term mortality (table 4).

**Table 4:** Univariate and multivariate predictors of long-term all-cause mortality

Variable	Univariate analysis			Multivariate analysis*		
	HR	95% CI	P value	HR	95% CI	P value
Demographics						
Age	1.04	1.03 to 1.06	<0.001	1.05	1.03 to 1.06	<0.001
Female	1.04	0.8 to 1.3	0.83	–	–	–
Indigenous	1.1	0.6 to 1.9	0.7	–	–	–
Rural	0.9	0.8 to 1.3	0.9	–	–	–
Precipitant of admission						
Adherence	0.9	0.5 to 1.6	0.76	–	–	–
Arrhythmia	0.6	0.4 to 0.8	0.002	0.8	0.6 to 1.3	0.4
Ischaemia	1.06	0.8 to 1.4	0.68	–	–	–
Infection	1.3	0.9 to 1.6	0.07	–	–	–
Cause of HF						
IHD	1.3	1.03 to 1.6	0.025	1.4	0.7 to 2.6	0.3
Cardiomyopathy	0.5	0.3 to 0.7	0.002	0.6	0.3 to 1.1	0.08
VHD	1.4	1.03 to 1.8	0.032	1.1	0.7 to 1.7	0.6
Comorbidities						
Atrial fibrillation/flutter	1.06	0.8 to 1.3	0.64	–	–	–
Stroke	1.2	0.9 to 1.7	0.22	–	–	–
CPD	1.3	0.9 to 1.6	0.055	–	–	–
CKD	1.7	1.3 to 2.1	<0.001	1.3	0.9 to 1.8	0.1
Diabetes mellitus	1.01	0.8 to 1.3	0.97	–	–	–
Hypertension	0.9	0.8 to 1.3	0.94	–	–	–
IHD	1.3	1.0 to 1.6	0.05	0.9	0.8 to 1.5	0.9
Specialty						
OMUs	1.6	1.3 to 2.1	<0.001	1.1	0.8 to 1.5	0.5
HF type (compared with HFrEF)						
HFpEF (EF≥50)	1.03	0.8 to 1.3	0.83	–	–	–
Medication						
ACEis/ARB	0.7	0.6 to 0.8	0.006	0.7	0.5 to 0.9	0.027
Aldosterone antagonist	0.65	0.5 to 0.84	0.001	1.2	0.8 to 1.7	0.3
β-Blockers	0.6	0.5 to 0.8	<0.001	0.8	0.6 to 1.1	0.2
Anticoagulant	0.8	0.6 to 1.01	0.06	–	–	–
Antiplatelets	1.0	0.8 to 1.3	0.99	–	–	–
Calcium channel blocker	0.9	0.7 to 1.4	0.95	–	–	–
Digoxin	1.0	0.7 to 1.4	0.96	–	–	–
Loop diuretics	1.0	0.8 to 1.3	0.99	–	–	–
Thiazide	0.8	0.5 to 1.2	0.29	–	–	–
Nitrate	1.7	1.2 to 2.4	0.003	1.3	0.9 to 2.0	0.2
Statins	0.8	0.7 to 1.1	0.15	–	–	–
Cardiac rehab	0.7	0.5 to 0.9	0.002	0.9	0.6 to 1.3	0.2
Pathology						
Peak troponin	0.98	0.97 to 0.99	0.04	0.99	0.98 to 1.0	0.1
Haemoglobin	0.98	0.97 to 0.99	<0.001	0.9	0.98 to 1.01	0.4
White blood cell	1.0	0.9 to 1.1	0.47	–	–	–
Platelet count	0.99	0.98 to 1.01	0.74	–	–	–
Albumin	0.93	0.9 to 0.95	<0.001	0.94	0.92 to 0.98	0.005
Creatinine at admission	1.001	1.001 to 1.002	<0.001	1.002	1.001 to 1.003	0.01

*Model included variables with P value of less than 0.05.

ACEis/ARB, ACE inhibitors/angiotensin II receptor blockers; CKD, chronic kidney disease; CPD, chronic pulmonary disease; GFR, glomerular filtration rate; HF, heart failure; IHD, ischaemic heart disease; OMUs, other medical units; VHD, valvular heart disease.

## Discussion

The main findings of this study are that even in a contemporary HF patient population following an index HF admission in a regional Australian setting: (1) this index HF admission is followed by high 1-year mortality and readmission (~66%); (2) guideline-directed medical therapy is underused in HFrEF (whether we used the new or the old guidelines), as is inpatient echocardiography and (3) 1-year all-cause mortality was 67% higher for the HFrEF group vs the HFpEF group (95% CI 1.14 to 2.44; p=0.009). To our knowledge, *this is one of the first Australian studies to report and compare long-term HF outcomes according to HF types*.

Due to the lack of universal consensus on HF patients with EF 40%–49%, we used 2018 Australian HF guidelines to separate HF into HFrEF (EF<50%) and HFpEF (EF>50%).^3^ The 2016 European guidelines defined a third group that is HF with mid-range EF (40%–49%)^4^ and the updated guidelines in 2021 renamed HF with mid-range EF as mildly reduced EF (HFmrEF).^20^ In this cohort, 82 patients (12%) would be classified as HFmrEF. However, a 10% difference in EF can be challenging and had significant variability between echocardiography reporter and modality of measurement.^21 22^ More interestingly, data from subgroups analysis of randomised controlled trials showed that angiotensin II receptor blockers, β-blockers and aldosterone antagonists reduce mortality and hospitalisation in HF patients with EF 40%–49% and this treatment should be considered according to the most recent HF guidelines.^20 23–25^ Despite previous data suggesting some HFpEF patients are in fact HFrEF with recovered EF,^18^ it is unlikely that patients with improved EF would be classified as HFpEF during hospitalisation since the majority of the patients in the current study had their echocardiography during their index HF hospitalisation.

The unadjusted rates of 1-year all-cause mortality in our study were similar in HFrEF and HFpEF (29% vs 23%, p=0.15). The high burden of HF is well recognised and HF mortality in this study is similar to the Australian nationwide rate.^26^ In the NSW HF snapshot, 12-month mortality rate was similar in those with EF≥50% (25%), patients with EF 40%–50% (25%), patients with EF 30%–39% (23%) and patients with EF<30% (27%).^27^ In addition, in a recent US registry data,^7^ the 5-year mortality was 75% for both HFpEF and HFrEF. In a large UK cohort study,^28^ there was only a modest increase in 1-year survival after index HF hospitalisation from 2000 to 2017 despite the emergence of multiple new lines of management over the same period. Interestingly, diagnosis at the time of HF hospitalisation was associated with less improvement in survival. Therefore, *early identification of HF patients in primary care might contribute to a reduction in hospitalisation and adverse outcomes*.^29^ Many factors might be responsible for high mortality in HF. Advancing age is an important predictor of mortality in many HF studies.^30^ In addition, high prevalence of comorbidities in HF patients might limit the choice of medication, which in turn can lead to worse outcomes.^31^ The prevalence of comorbidities in this study was similar to other national and international studies.^12 13 32 33^ Ischaemic heart disease was the main cause of HF and the prevalence of ischaemic heart disease was greater in HFrEF patients, once again similar to NSW HF snapshot.^13^

One of the other key findings of this study is the low utilisation of guideline-directed medical therapies in HFrEF, despite the lack of contraindications. Even among patients without chronic kidney disease, 45% were not on ACE inhibitors/angiotensin II receptor blockers. Updated Australian HF guidelines in 2011 recommended the use of ACE inhibitor/angiotensin II receptor blockers, β-blockers and aldosterone antagonist,^17^ however, only 27% were on this recommended combination therapy. This figure is concerning, especially given the NSW HF snapshot study showed similar figures,^13 27^ as did two other Australian studies.^12 32^ Interestingly, our sensitivity analysis showed a significant increase in the prescription of medication on discharge, compared with admission, but the rate was still low (online supplemental materials). Unfortunately, the adherence and rate of utilisation of these medications after discharge is unknown. Randomised controlled trials showed a clear benefit from these therapies in reducing HF morbidity and mortality.^3 34^ A recent study showed adherence to guideline treatment within 60 days postdischarge is associated with lower 1-year mortality in older HF patients.^35^ Thus, *more effort and resources should be directed to improve utilisation and adherence to the guidelines*. In addition, with more emerging evidence for the benefit of ACE inhibitor/angiotensin II receptor blockers, β-blockers and aldosterone antagonists in HF patients with EF between 40% and 49%,^20^ healthcare providers should be educated about these updates. As shown in this study, the number of patients eligible for these therapies increased from 145 to 193 patients (25% increase).

Another important finding of the study is that one-third of the patients did not have inpatient echocardiography or echocardiography within 1 year of admission. Australian, European and American guidelines strongly recommend echocardiography for patients with HF.^3 4 18^ In the NSW HF snapshot study, only 7% did not have inpatient echocardiography.^13^ However, some studies might be biased to accommodate inpatient echocardiography for trial patients. Most Australian studies do not differentiate between HF types, as they are reliant on discharge ICD-10 coding which often defaults to HF not otherwise classified. In a systematic review of 13 Australian studies to report HF mortality and readmission, only three trials reported HF types.^26^ Only a minority of patients had brain natriuretic peptide (BNP) levels in this study, therefore we did not report it in the analysis. The lack of echocardiography services for a significant proportion could highlight the critical area of need in regional Australia with limited access to essential services.

Our demographic data are consistent with prior studies and thus the conclusions can be generalised to the broader general population. The median age of HF patients was 80 years (mean age 77 years) which is similar to the NSW HF snapshot study. Similarly, HFrEF patients were younger than HFpEF patients in both studies,^13^ as well as in other studies.^7 33^ Males were over-represented in HFrEF patients compared with HFpEF and this is also consistent with national and international data.^7 27 33^ One of the possible explanations is that ischaemic heart disease is more common in males and two-thirds of HFrEF is related to ischaemic heart disease.^13 36 37^

The propensity-matched scoring allowed us to make the HFpEF and HFrEF groups comparable by other demographic and etiological factors. This demonstrated a 67% higher 1-year all-cause mortality was in HFrEF than HFpEF group. In a recent community prospective analysis from New Zealand and Singapore,^33^ HFrEF patients also had higher mortality than HFpEF and mid-range HF patients, but there are still conflicting data in the

literature regarding outcomes by HF types. Other large long-term follow-up studies showed similar death rates in HFpEF and HFrEF patients.^38^ On the other hand, in the MAGGIC meta-analysis of 28 observational HF studies,^39^ HFrEF had higher mortality than HFpEF. Age, rurality and stroke were predictors of poor outcomes, while admission for arrhythmia, ACE inhibitor/angiotensin II receptor blocker use, high albumin and digoxin use were associated with better outcomes. Overall, long-term mortality was very high in our HF patients and was similar in HFrEF and HFpEF cohorts. Being on guideline medical therapy was a predictor of lower long-term mortality even after adjusting for multiple variables. In addition, regional and rural patients may be disadvantaged by few factors which can lead to poor outcomes.^40 41^ Our cohort is unique in that it is geographically and socioeconomically different from metropolitan areas in the capital cities of Australia, and it represents the regional NSW. *HF programmes in regional Australia need to address these areas of unmet clinical need to improve long-term outcomes*.

This study had several limitations. It is an observational retrospective study, and the reliability of study conclusions is dependent on the accuracy of the medical records. However, to optimise our accuracy, every patient’s medical record was reviewed individually, and discrete data points were extracted manually by a medical practitioner. In addition, we performed a sensitivity analysis using HF as the principal diagnosis, which showed similar results (see online supplemental data) and it has been shown that HF as a principal diagnosis has a positive predictive value of 99.5.^42^ Another limitation is that one-third of the patients did not have inpatient echocardiography and therefore were unable to be classified as HFpEF or HFrEF. Moreover, there are no data on ECG, the presence of left bundle branch block and only one patient had cardiac resynchronisation therapy, making it difficult to comment on appropriateness of utilisation of this therapy further. The lack is ECG data is an important limitation because ECG abnormalities can indicate advanced HF and provide prognostic information.^43^ However, the long-term follow-up of the study would reduce the impact of this limitation. While a limitation, this *highlights another critical area of the policy-practice gap-underutilisation of guideline-recommended CV investigations to aid HF diagnosis*! While we cannot dissect the reasons for it, lack of access especially for patients living regionally and rurally is likely to be a significant contributor. Furthermore, the medication on discharge does not reflect future adherence and postdischarge dispensing. Moreover, data on BNP and N-terminal pro-BNP were lacking: these are used in a very limited fashion in Australia due to the lack of Medicare reimbursement for these tests. Finally, details of contraindications to guideline treatment (other than renal function) in HFrEF patients were not available.

In conclusion, despite ongoing improvements in the management of HF, mortality and morbidity remain high in HF patients, who are generally elderly and have multiple comorbidities. This finding is consistent across HF types, but HFrEF may carry a worse prognosis than HFpEF in our contemporary metropolitan, regional and rural Australian cohort. These poor outcomes could be at least in part explained by poor adherence to guideline-directed HF therapy and underutilisation of echocardiography, which in turn would contribute to further underuse of guideline-directed therapies. Concerted efforts need to be made to (1) improve adherence to guideline-recommended HF therapies; (2) improve access to and utilisation of echocardiography and natriuretic peptide testing for HF patients, especially in regional and rural populations and (3) develop tailored and dedicated HF management programmes for regional and rural populations, especially targeted to primary care and transitions of care.

## Data Availability

All data relevant to the study are included in the article or uploaded as supplementary information.
