# Supplementary material for: Patient characteristics, short-term and long-term outcomes after incident heart failure admissions in a regional Australian setting
Source: Open Heart. 2022 May 31;9(1):e001897. doi: 10.1136/openhrt-2021-001897 (PMC9157343; doi:10.1136/openhrt-2021-001897)
Supplement: Supplementary data [file openhrt-2021-001897supp001.pdf]

SUPPLEMENTARY MATERIALS

Patient Characteristics and Long Term Outcomes of Incident Heart Failure Admissions in a Regional Australian Setting

This is a cohort study of heart failure patients admitted to hospitals in HNE LHD in the calendar year 2014. The sample includes patients admitted with a diagnosis of heart failure in the 1st 4 diagnoses. To capture *incident* heart failure admissions only, patients with a prior admission for heart failure in the preceding 10 years (from Jan 2005 to Dec 2013) were excluded. The cohort is a group of patients who were admitted in 2014 to John Hunter Hospital & Tamworth Rural Referral Hospital in 2014 (n=686).
The goals of the study are identification of the main 2 types of heart failure which are heart failure with reduced ejection fraction (HFREF) and heart failure with preserved ejection fraction (HFPEF), comparing the outcomes of these 2 groups in terms of mortality and re-hospitalisation, and to the adherence to guidelines for treatment of HFREF.

Propensity matched score:

Outcomes:

1- Death or readmission within 1 year.

Logistic regression with IPTW was used to assess the difference between heart failure groups on death or at least 1 readmission within 1 year. Odds ratios with 95% confidence intervals and p-values are provided.

2- Time to death - all causes.

A Cox regression with IPTW was used to assess the difference in time from admission to death between heart failure groups. Patients were censored at the 1 year follow up time. Hazard ratio with 95% confidence intervals and p-values are provided along with plots of survival and cumulative hazard functions.

3- Time to all causes readmission.
4- Time to heart failure readmission.

A Cox regression including death as a competing risk with IPTW was used to assess the difference in time from admission to first readmission and first heart failure readmission between heart failure groups. Patients were censored at the 1 year follow up time. Cumulative incident plot, hazard ratio with 95% confidence intervals and p-values are provided.

Patients with a `Not recorded' heart failure and any missing covariates were excluded from the analysis for outcomes, time to death, time to readmission and time to heart failure readmission. Statistical analyses were programmed using SAS v9.4 (SAS Institute, Cary, North Carolina, USA).

Results:

the IPTW has increased the balance of selected covariates across heart failure groups.
Comparison of IPTW and non-weighted covariates by heart failure groups.

- Table S1: Comparison of IPTW and non-weighted covariates by heart failure groups.

		Non-weighted			*IPTW Weighted*		
Variable	*Statistic*	*Heart failure (EF<50%)*	*Heart failure (EF>=50%)*	*P-value*	*Heart failure (EF<50%)*	*Heart failure (EF>=50%)*	*P-value*
Age at adm	mean (SD)	75 (13)	77 (11)	0.1017	76 (13)	76 (11)	0.8146
	median (Q1, Q3)	77 (66, 84)	79 (70, 85)	0.1587	79 (69, 86)	78 (70, 84)	.
Hemoglobin	mean (SD)	123.5 (23.4)	119.6 (20.7)	0.0720	121.8 (23.3)	121.7 (21.5)	0.9493
	median (Q1, Q3)	125.0 (111.0, 138.0)	119.0 (106.0, 133.0)	0.0187	120.0 (111.0, 136.0)	122.0 (107.0, 135.0)	.
Haematrocrit	mean (SD)	0.4 (0.1)	0.4 (0.1)	0.3363	0.4 (0.1)	0.4 (0.1)	0.9804
	median (Q1, Q3)	0.4 (0.3, 0.4)	0.4 (0.3, 0.4)	0.0140	0.4 (0.3, 0.4)	0.4 (0.3, 0.4)	.
WCC	mean (SD)	10.3 (4.3)	9.8 (4.4)	0.2216	9.9 (4.2)	10.0 (4.3)	0.9737
	median (Q1, Q3)	9.5 (7.5, 12.6)	8.7 (7.0, 11.7)	0.0748	9.0 (7.1, 12.3)	9.0 (7.2, 11.9)	.
Neutrophils	mean (SD)	7.8 (3.8)	7.2 (3.7)	0.1156	7.5 (3.5)	7.4 (3.8)	0.8751
	median (Q1, Q3)	7.2 (5.1, 9.5)	6.3 (4.8, 8.9)	0.0604	7.0 (5.0, 9.1)	6.4 (4.8, 9.0)	.
Lymphocytes	mean (SD)	1.5 (0.9)	1.4 (0.9)	0.2184	1.5 (1.0)	1.5 (0.9)	0.9160
	median (Q1, Q3)	1.3 (0.9, 2.0)	1.2 (0.8, 1.7)	0.1398	1.3 (0.9, 1.9)	1.3 (0.9, 1.8)	.
Monocytes	mean (SD)	0.7 (0.3)	0.7 (0.3)	0.2077	0.7 (0.3)	0.7 (0.3)	0.7632
	median (Q1, Q3)	0.7 (0.5, 0.9)	0.6 (0.5, 0.8)	0.1724	0.6 (0.5, 0.8)	0.7 (0.5, 0.8)	.
Basophils	mean (SD)	0.0 (0.1)	0.0 (0.1)	0.0333	0.0 (0.0)	0.0 (0.1)	0.5850
	median (Q1, Q3)	0.0 (0.0, 0.1)	0.0 (0.0, 0.0)	0.0034	0.0 (0.0, 0.0)	0.0 (0.0, 0.0)	.
eGFR adm	mean (SD)	58.7 (22.7)	51.8 (23.1)	0.0025	53.5 (24.4)	54.5 (23.2)	0.6836
	median (Q1, Q3)	63.0 (41.0, 78.0)	52.0 (35.0, 70.0)	0.0026	54.0 (32.0, 75.0)	56.0 (36.0, 73.0)	.
eGFR disch	mean (SD)	57.3 (22.6)	51.9 (22.7)	0.0162	52.9 (24.1)	53.8 (22.9)	0.7016
	median (Q1, Q3)	58.0 (42.0, 77.0)	49.0 (36.0, 70.0)	0.0122	54.0 (36.0, 73.0)	52.0 (37.0, 74.0)	.

		Non-weighted			*IPTW Weighted*		
Variable	*Category*	*Heart failure (EF<50%)*	*Heart failure (EF>=50%)*	*P-value*	*Heart failure (EF<50%)*	*Heart failure (EF>=50%)*	*P-value*
Sex	Female	63 (34%)	117 (53%)	0.0002	82.6 (44%)	97.2 (44%)	0.9919
	Male	122 (66%)	105 (47%)		104.9 (56%)	123.1 (56%)	
Cause IHD	No	81 (44%)	131 (59%)	0.0022	100.1 (53%)	115.7 (52%)	0.8551
	Yes	104 (56%)	91 (41%)		87.4 (47%)	104.7 (48%)	
Cause HTN	No	105 (57%)	91 (41%)	0.0015	87.5 (47%)	104.0 (47%)	0.9137
	Yes	80 (43%)	131 (59%)		100.1 (53%)	116.4 (53%)	
CoMorbidity CKD	No	129 (70%)	126 (57%)	0.0071	111.1 (59%)	136.7 (62%)	0.5650
	Yes	56 (30%)	96 (43%)		76.4 (41%)	83.7 (38%)	
CoMorbidity HTN	No	84 (45%)	62 (28%)	0.0003	65.5 (35%)	77.0 (35%)	0.9983
	Yes	101 (55%)	160 (72%)		122.0 (65%)	143.4 (65%)	
CABG	Before index admission	36 (19%)	23 (10%)	0.0062	26.6 (14%)	30.1 (14%)	0.9810
	During index admission	6 (3.2%)	2 (0.9%)		3.7 (2.0%)	3.9 (1.8%)	
	Not recorded	143 (77%)	197 (89%)		157.3 (84%)	186.3 (85%)	
PPM	No	162 (88%)	206 (93%)	0.0745	170.5 (91%)	201.6 (91%)	0.8388
	Yes	23 (12%)	16 (7.2%)		17.0 (9.1%)	18.8 (8.5%)	
Calcium	No	161 (87%)	184 (83%)	0.2467	158.7 (85%)	187.1 (85%)	0.9456
	Yes	24 (13%)	38 (17%)		28.8 (15%)	33.3 (15%)	
Diuretics	No	41 (22%)	56 (25%)	0.4702	45.0 (24%)	52.6 (24%)	0.9792
	Thiazide/Loop	144 (78%)	166 (75%)		142.5 (76%)	167.7 (76%)	

Sensitivity analysis

All consecutive index HF admission to John Hunter Hospital with HF being the principal diagnosis (n=199), as opposed to one of the top four diagnoses. Forty-four patients (22%) were HFrEF, 49 patients (25%) were HFpEF and the rest (53%) were non-classified.

- Table S2: Demographics and HF features of the sensitivity analysis cohort.

	HFrEF (n=44)	HFpEF (n=49)	Non-classified HF (n=106)	Total (199)	P-value
**Demographics**					
Age in years, median (IQR)	80 (70-86)	81 (75-85)	84 (78-88)	82 (75-87)	0.01
Female, number (%)	14 (32)	22(45)	58 (55)	94 (47)	0.035
Indigenous, number (%)	-	-	-	2 (1)	-
Rural, number (%)	-	-	-	15 (8)	-
Length of stay in days, (IQR)	5.5 (3-10)	5 (3-12)	4(2-6)	4 (2-8)	0.01
**Precipitant for admission, number (percentage)**					
Adherence	-	-	-	10 (5)	-
Arrhythmia	-	-	-	50 (25)	-
Ischemia	-	-	-	22 (11)	-
Infection	-	-	-	36 (18)	-
Other/unknown	-	-	-	10 (5)	-
**Cause of HF, number (percentage)**					
IHD	24 (55)	18 (37)	47 (44)	89 (45)	0.24
Cardiomyopathy	-	-	-	14 (7)	-
VHD	-	-	-	37 (19)	-
**Co-morbidities, number (percentage)**					
Atrial Fibrillation / Flutter	23 (53)	23 (47)	49 (46)	95 (48)	0.7
Stroke	-	-	-	32 (16)	-
CPD	13 (30)	20 (41)	36 (34)	69 (35)	0.5
CKD	13 (30)	23 (47)	45 (42)	81 (41)	0.2
Diabetes mellitus	14 (32)	13 (27)	29 (27)	56 (28)	0.82
Hypertension	24 (56)	38 (78)	79 (75)	141 (71)	0.039
IHD	23 (52)	19 (39)	47 (44)	89 (45)	0.42
**Admitting Speciality, number (percentage)**					
Cardiology	25 (57)	27 (55)	38 (36)	90 (45)	

OMUs	19 (43)	22 (45)	68 (64)	109 (55)	0.018
**Outcomes, number (percentage)**					
1-year death	14 (32)	10 (20)	29 (27)	53 (27)	0.4
1-year all cause readmission	20 (45)	29 (59)	55 (52)	104 (52)	0.4

**Guideline Medication, number (percentage) in HFrEF (n=44)**			**P value**
	On admission	On Discharge	
ACEis / ARB, n/total (%)	21/41 (52)	30/44 (68)	0.1
B-Blocker, n/total (%)	22/41 (54)	34/44 (77)	0.02
Aldasterone antagonist, n/total (%)	2/41 (5)	18/44 (41)	<0.001

HF: Heart Failure. IHD: Ischemic Heart Disease. CPD: Chronic Pulmonary Disease. CKD: Chronic Kidney Disease. ACEis / ARB: Angiotensin-Converting Enzymes inhibitors / angiotensin II receptor blockers. OMUs: Other Medical Units. IQR: Interquartile range.
